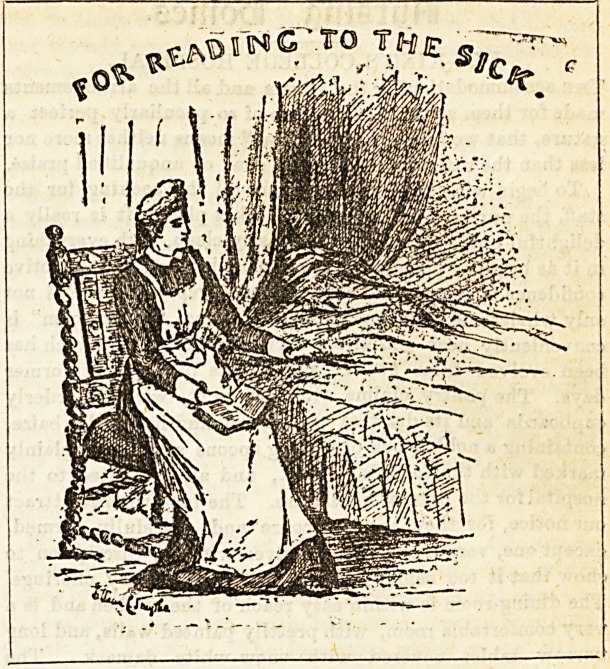# The Hospital Nursing Supplement

**Published:** 1892-05-07

**Authors:** 


### "The Hospital" Nursing Mirror.

Being the Extra Nursing Supplement of "The Hospital" Newspaper.

Contributions for this Supplement should be addressed to the Editor, The Hospital, 140, Strand, London, W.C., and should have the word "Nursing" plainly written in left-hand top corner of the envelope.

### En Passant.

LONDON SCHOOL OF MEDICINE FOR WOMEN.—An arrangement has been made between this school and the Royal Free Hospital that the hospital shall receive 4 per cent. upon all fees paid by students in order to cover the expenses incurred by the hospital on behalf of the students.

CORRESPONDENTS.—Will some of our correspondents please notice our rules which we insert from time to time, notably the one which states that we cannot take notice of any communication, whether letter, note, or query, unless it be accompanied by the writer's full name and address. Also a private letter is sent in reply in *urgent* cases, but then a stamped envelope must be enclosed.

WOOLWICH DISTRICT NURSING.—A most successful annual meeting of this society gave a report of a year's good work. One nurse in a large district succeeded in averaging six visits a day, and we are glad to hear that a second nurse is now sharing her labours. This society is affiliated to the Q.V.J.I., and the central body has given £40 to the support of the second nurse. Mr. Warrington Howard, F.R.C.S., surgeon to St. George's Hospital and lecturer to the Metropolitan Association for Training District Nurses gave an interesting address on the advantages of district nurses firstly to the sick poor, secondly to their medical attendants, and thirdly to the community.

NURSES FOR INDIA.—A correspondent who has spent many years in India, writing on the letters which have appeared about a band of nurses starting for India, says: "I personally know of several nurses who, on completion of their training, have started out here on their own account, and, I am sorry to say, most of them have been disappointed at the realisation of their hopes not coming up to their anticipations. . . . A nursing band to emigrate to India and meet with success would require a strong committee of well-wishers to back them up, and a still stronger supply of funds. There are many folk who cannot afford the luxury of a private nurse (Rs.5 a day, as a rule), who can be nursed at the Government Civil Hospitals through any illness for whatever payment their pocket can afford. However, "Nothing venture, nothing have," and if a band of women can overcome all these trials and difficulties they will have my warmest admiration." This shows that this Indian scheme needs much deliberation. Colonel Butler Hamilton proves to us that the need of nurses does exist, and we shall be glad to hear of any more evidence on the question. There can be no doubt that this would have to be a large enterprise, financially and otherwise, but we can only hope that ere long we shall hear of some substantial move being made towards it.

SAMARITAN SOCIETY AT ST. THOMAS'S.—We are sorry to see that the receipts of this fund have fallen off to the amount of £124 compared with those of the previous year. An increased expenditure is due to the severity of the cases which have been kept a longer period at convalescent homes or elsewhere in order to secure complete restoration of health. 1852 was the year in which this excellent Society started on its career of usefulness, and as the years go on it is necessary to extend its sphere. "The best mode of doing this," the report modestly suggests, "would be by founding a convalescent home attached to the 'hospital' with sufficient accommodation to relieve the pressure at present existing upon the beds at the hospital." The work of any of these "after care" societies can never be too fully appreciated. After the careful treatment and long weeks of good nursing, how often does a patient feel fit to go straight out of hospital and begin his fight in the world afresh? It needs a kindly hint now and again to remind a very good-hearted public that a hospital of itself cannot always manage to put the finishing touch to a sick person's recovery, but with the aid of a well-filled purse it can and does enable its patients to get that longed-for sight of the sea or scent of the pines, which dispels the feeling of lassitude and weariness, and gives new hope without which recovery can never come. We hope some readers may consider the reduced funds of this Samaritan Society; a copy of the report will show what it does for its sick folk.

THE CARE OF THE INSANE IN NEW YORK CITY.—The Committee appointed by the Mayor to investigate this matter recommends that the insane of the city be not given over to the care of the State, that the maintenance allowance be increased, and the Medical Superintendent be given a larger salary, and fuller powers respecting his assistants. The *New York Medical Record* mentions the case of the insane, who are not paupers. There is a very large and deserving class who do not want to send their friends to a pauper institution, and who cannot afford the magnificent payment required by a private asylum, but who could pay about 15s. or 16s. a week. There remain only the State institutions for them to go to, and if the city is exempted, as it is to be, from its State tax for the insane, this will be impossible.

LOWESTOFT HOSPITAL.—We are sorry to hear of troubled waters at this institution. The Committee, because they had an arrangement in 1887 which was satisfactory, are annoyed because the Matron wishes other arrangements now. There were then two fully-trained nurses, one for the men's wards and one for the women's; now the Matron wishes the arrangement to consist of a sister for the female ward and a staff nurse for the male, the one subordinate to the other to avoid frictions, which occurred when the authority was equal. The Committee supported the proposition, but resolved to adhere strictly to the 1887 arrangement (this is interesting; how did they manage to do both?). A resolution passed by the Committee lately tends, in the opinion of the President, Captain Larkings, and Dr. Walker, one of the surgeons, to tamper with the rules giving the Matron full authority, and the unfortunate result of all this is the resignation of the Matron and the President.

THE CARE OF THE "FEEBLE-MINDED."—Our readers will be glad to hear that already an energetic committee at Birmingham has secured two cottages near Barnet Green for young and innocent girls who are mentally defective to a certain extent or are, as they have been aptly described, deficient in self-protecting qualities. The idea is to start laundries and other homes of industry for the girls to work in, and to provide cottage homes presided over by a "Mother," who will have to be a carefully selected person of unquestionable character. It must be remembered that detention is impossible; the scheme can only work on a voluntary system, and it is believed that once in a home the girls will be glad to remain. The whole will be under the inspection of the Local Government Board. It is hoped that remunerative work will enable these girls to pay for part of their maintenance, and it is expected that the boards of guardians will pay for the cases sent by them, and in other cases the relatives and friends will be responsible. Space does not allow us to give the full detail of an admirably considered scheme, but we hope that those who see this paragraph will make the movement as widely known as possible. It is a question of public importance, and at present the lack of care of these girls constitutes an evil which few will perhaps realise. Miss Stacey, 30, Calthorpe Road, Birmingham, would send full details of the proposed arrangement to anyone interested.

### Ventilation, Disinfection, and Diet.

By P. Caldwell Smith, M.D.

### IV.—ARTIFICIAL VENTILATION—EXTRACTION—PROPULSION.

There are other forms of inlets used, as, for example, the Sherringham Ventilator. These are of different sizes, but a good size for an ordinary room is 9 in. by 4 in. These are placed in the wall, and open by means of a cord, the air being directed upwards. They are very good inlets, but the wind may blow through them, and they may be converted into outlets.

Another form of inlet used, and one which can be applied to any ordinary room is what is called the Hincke's Bird method. An ordinary window is raised one or two inches, and a piece of board is inserted, the air coming in between the sashes, and being thus directed upwards. Many modifications of this are in use, one of which is to make the sill deeper, and the lower bar of the sash deeper also, so that when the window is raised there is a space between the sashes, but no air can enter below, owing to the depth of the lower bar. This modification of course, has to be fitted when a house is building, but the simpler method can be used in any sick room, and ought to be one of the principal means used for ventilating it. There are several other methods of ventilating a room with warmed air besides the use of hot-water pipes or the use of mechanical ventilation, but the only one I think necessary to mention, is one which, without involving much constructive detail, ought to be applied to every occupied apartment, whether in private houses or in hospitals where open fires are used. I refer to Galton's ventilating fire-place. By far the greater amount of heat produced by an ordinary open fire-place goes up the chimney, only about one-fifth being available for heating the room. Some fire-places, of course, cause more heat to be utilized, but all fail to utilise even one-third of the heat generated. Again, the brighter fire we keep up in a room the greater will be the draught between any inlet, which is in most cases the door, and the fire, thus chilling the feet of those sitting in a line between these. Sir Douglas Galton, recognising this, states that "the only way to prevent draughts is to adopt means for providing fresh warmed air to supply the place of that removed." He also, in introducing his fire-place, saw that the best place to introduce warmed air was above the chimney-piece, or at any spot between that and the top of the room.

Fresh air is admitted from the outside to an air-chamber at the back of the grate, where it is warmed, and introduced into the room about two or three feet from the ceiling.

There are several important details in the construction of the grate itself, but these are too technical to enter into. Sir Douglas Galton, however, states that they were designed to render the combustion of the coal more perfect. The only objection that I have heard of regarding these is, that if they are the only means used for admission of air, the air is apt to be burnt or made too dry; but by utilising the window occasionally as an inlet, and regulating the fire, this can be to a large extent prevented. I cannot do better than give Sir Douglas Galton's own words regarding the merits of the stove:—

"(1) That it ventilates the room.

"(2) That it maintains an equable temperature in all parts of the room, and prevents draughts.

"(3) That the heat is thrown into the room better than from other grates.

"(4) That the fire-brick lining prevents the fire from going out, even when left untouched for a long time, and prevents the rapid changes of temperature which occur in rooms in cold weather from that cause.

"(5) That it economises fuel, partly by making use of the spare heat, which otherwise would all pass up the chimney, and partly by ensuring by its construction a more complete combustion, and thereby diminishing smoke.

"(6) That it prevents smoky chimneys by the ample supply of warmed air to the room, and by the draught created in the neck of the chimney."

George's Calorigen may also be used as a heating and ventilating agent, but in this case gas is used, the products of the combustion being at once carried to the open air.

A few remarks regarding outlets. These should always be placed at the top of the room, but in ordinary dwelling houses we seldom see them, unless in the form already spoken of, viz., Arnott's valve placed in the chimney. The fire-place itself is, in by far the greater number of houses, the only outlet for the foul air. In hospitals, schools, &c., where no mechanical system is used, ridge ventilators are necessary, as Buchan's, Boyle's, &c., which carry off the foul air by means of the wind blowing across them.

We now enter shortly upon the subject of artificial ventilation, and there are two main divisions of this, viz. (1) Ventilation by extraction, or what is called the vacuum method; and (2) Ventilation by propulsion, or plenum method. In the former, ventilation by extraction, the extracting power is produced either by heat or by a fan. An ordinary chimney is a good example of extraction by heat. The number of cubic feet of air passing up the chimney varies, but it may be roughly stated as from 3 to 6 feet per second, or from 10,800 to 21,600 feet per hour. Thus we see that an ordinary chimney, with a good fire burning, ventilates a room sufficiently for four or five persons. When the fire draws well, all the other openings in the room become inlets, thus showing the power of this method of ventilating. The Houses of Parliament are ventilated by means of heat applied as in the case of the ordinary chimney. The fresh air taken from the adjacent areas is admitted to the basement of the building, warmed by being passed over pipes heated by steam, and passed into a chamber below the floor, from which it is admitted to the House itself through gratings in the floor covered by matting. The ceiling of the House is made of glass, in which there are numerous openings into a chamber at the roof, and from this a shaft is led to the foot of the tower, where a fire is kept burning. It is said that over one and a-half million cubic feet per hour can be introduced into the House, this giving an average of 2,000 cubic feet to each individual when it is full. For hospitals there are several objections to this method: (1) The draught is unequal, due to the fact that the fire cannot always be kept at the same height; (2) if applied to more than one room or ward, those nearest the shaft are more quickly acted on than those at a distance; (3) smoke sometimes regurgitates from the shaft through the tubes into the rooms; (4) in a hospital ward especially, fresh air may be drawn from badly ventilated corridors, or from the lavatories. Ventilation by means of extracting fans is one of the best forms of applying this principle, and it is coming more into use. In the Hillhead public school one of these extracting fans is employed, and works very satisfactorily. The late Professor Carnelly found that in this school the air was very pure, and that the temperature throughout the whole school remained pretty constant at 58° F. The fan is placed at the top of the school near the roof, and tubes lead from the different rooms to it, while fresh air, properly warmed, is introduced at suitable places. The other mechanical system used is ventilation by propulsion. By means of a fan the air is forced into the rooms or wards through suitable tubes after being purified and heated. The new Victoria Infirmary is heated and ventilated in this way, and is said to give very satisfactory results.

(*To be continued.*)

### The Nursing of the Insane.

By a Medical Officer.

### WHY TRAINING IS NECESSARY.

(*Continued from page* xxxi.)

In addition to the sorrow naturally experienced when a relative is afflicted with a disease which unfits the sufferer to mingle with his friends, and requires him to be separated from wife and child, there is a feeling of shame as if the family honour had been stained. No doubt a glimmering of the truths of heredity plays some part in this, but the foundation is laid by the old-world idea that insanity should be punished, and the sufferer immured within the walls of the asylum. Ignorant of the fact, or ignoring it, if they are aware of it, that the sooner such a patient is placed under treatment the better are his chances of recovery, the friends allow him to remain at large until he can be no longer borne with. When he has become a source of anxiety, danger, or ridicule, then he is sent to be put under the medical treatment which might have been efficacious months before. The enlightened intelligence of those who make laws for our guidance still further confirm these lingering superstitions. The Lunacy Act fences round the sending of a lunatic to an asylum with so many formalities that it is only natural the troublesome process is put off until the last moment. It ought to be as easy to place a sufferer from brain disease under proper treatment as to send a man with a broken leg to an infirmary, and the nurse who tends to the one should be as carefully trained as she who looks after the other. To alter the Lunacy law is a task which will require time; to see that his nurses are instructed lies within the power of every Superintendent. The brightness and cheerfulness of our asylum wards are worthy of all praise; the kindness and forbearance of the nurses show as light against darkness, when compared with the "keepers" of the past; but more is yet to be done. However well-intentioned a nurse may be, her efforts cannot bear full fruit unless they are directed by knowledge. Their powers of observation must be trained, they must be taught the essentials of their calling—and how can they learn if they have not a teacher?

No public body would to-day build a large hospital, and have attached to it a home for incurables, without making provision for a supply of skilled attendants on the sick. If they neglected this important duty public opinion would compel them to undertake it. Unfortunately, with regard to the insane, public opinion requires to be educated. We still pay the penalty for the errors of the past. The fathers ate sour grapes, and the children's teeth are set on edge. The girl is taken from household duties in county or in town; the man is taken from the plough or the workshop, and is at once placed in charge of the insane. This is unjust to the insane themselves, and to those who pay for their care and maintenance, while it is grossly unfair to the physicians who are responsible for their treatment.

Our county councils are making efforts for the spread of technical education in their districts. Here is a case in which a body of their own servants is calling for instruction, which it is to the advantage of their employers to provide. It is in the power of Superintendents and the asylum committees to raise the status of the women employed in their wards, and at the same time make more efficient servants of them.

### Wants and Workers.

A lady kindly offers to send us the following surgical appliances for the use of some poor persons in a hospital or elsewhere. An artificial arm for the left side; it laces on the arm above the elbow and below the shoulder, each finger has joints in it and there are two forks which fix into a spring in the palm of the hand, which can be used for cutting food. Three joints in the elbow enable anyone to put the arm at different angles. The former wearer of the arm could knit or sew with it. Made by Wood of York. Also some supports worn by a boy of twelve; they are strapped on the thigh and leg and ended by being slipped into boots, these latter are worn out. Also a pair of irons which have been used for a boy who had the tendons at the back of the heels cut, these screw up as the case proceeds. There are also a few appliances for deafness.

### SICK AND AFFLICTED.

Loss of health is acknowledged on all sides to be the greatest trial man has to bear; to be sick and afflicted is indeed a most grievous experience. The words do not so much speak to us of sudden attacks of illness, which, though sharp and dangerous for the moment, pass away; or of an accident which may be remedied by care and time, but of that long weakness and wearing pain of which none can foresee the end, which makes life a weariness and admits of no alleviation. By it we seem shut out from all joy, hope, and even desire of change, we can do nothing for ourselves, nothing for our Master, the present fills all our thoughts, past joys are forgotten, and future ones are out of the question, we see nothing but an ever-increasing torment, a dread lest we shall not be able to endure to the end. But suffering, though inevitable in some way to all, need not be hopeless. The Light of Life, who came down from Heaven to die for us, taught us by His example how to bear our woes, and we will take the Cross for glory and for guide, and sitting at Christ's feet in obedience, and humility learn the lessons He teaches.

Whether a life of pain is gain or loss is questioned by some, but we may rest assured that it is the one thing which comes straight from God's hand, and by it we can follow Him more closely. Healthy life, love, and all other earthly joys come equally from Him; alas! we are so apt to forget the Giver in the gifts, but we cannot forget the Hand that chastens while it holds us and presses us so firmly that we cannot slip from under its clasp. Why we suffer is hidden from us in God's wisdom; it may be for His glory, and if we bear it bravely we shall, when this life is ended, be greeted with "Well done, thou good and faithful servant, enter thou into the joy of thy Lord."

Anyway, suffering is for our own purification and strengthening, and we must not be impatient and so let the cross slip. Here we are born to sorrow as the sparks fly upwards; hereafter will be the perfection of all our service, our love, our praise, our work for God. Our perfecting in Him begins on earth in drinking from the cup which was drained to its dregs by our dear Lord. We may see Him now if we will holding this cup towards us and encouraging us to taste it. "Drink with me," He says, and can we refuse? No; we will hold fast His hand, though the nails pierce us too. Where He wounds, there will He heal. Let us set our minds to suffer in His spirit, to follow in His footsteps though the road be sharp and thorny, so shall we be like Him, perfect through suffering.

### Nursing Homes.

### IV.—KING'S COLLEGE HOSPITAL.

The accommodation for the nurses and all the arrangements made for them at this hospital are of so peculiarly perfect a nature, that we feel a "description" means neither more nor less than the utterance af a great deal of unqualified praise.

To begin with the kitchen, where all the cooking for the staff, the nurses, and the patients takes place; it is really a delightful one. So airy, so spotlessly clean, with everything in it as brightly polished as it can possibly be; an instinctive confidence is engendered that the food prepared there is not only wholesome, but appetising. The cool "milk-room" is conveniently near and also a serviceable scullery, which has been evolved from two of the useless passages of former days. The pantry claims attention next, with its orderly cupboards and its drawers partitioned and lined with baize, containing a noble stock of shining spoons and forks plainly marked with the initials K.C.H., and all presented to the hospital for the nurses' special use. The tea-urns also attract our notice, for they are of bronze and gracefully formed, except one, very handsome, of silver with an inscription to show that it too belongs to the list of voluntary offerings. The dining-room is within easy reach of the kitchen and is a very comfortable room, with prettily painted walls, and long narrow tables covered with snow-white damask. The Matron dines daily with the nurses and Sisters and the food provided is very varied and excellent without being extravagant, whilst the supply of milk for those who like to drink it is unlimited.

There is an ingenious contrivance fixed under the dining tables to form a kind of sloping footrest for the nurses which appears a decidedly original idea. A second smaller but equally pleasant room is used for the night nurses' breakfasts, &c., and for the other nurses lunches of hot milk and coffee, in fact it is a "relief" room to save any over-pressure from the meals which follow so rapidly as almost to over-lap at certain hours.

The staff nurses are lodged in cubicles which are very airy and comfortable, with fine old oak flooring as a distinctive characteristic. Over these cubicles is the famous roof, the raising of which was a grand and ambitious improvement, meaning, in fact, the conversion of some very close and dull little apartments into these present satisfactory sleeping-rooms, and, the cost being completely defrayed, who will do aught save admire the results of this heroic undertaking!

A warmed corridor shut off by double baize doors is devoted to the nurses' use for "recreation," in any form in which they elect to amuse themselves—and the ever-welcomed kettle is here always boiling for any occasional tea making. The room where the Home Sister gives her classes is on the same floor and in it, too, the lectures are given by various members of the medical staff.

The paying probationers have each a very pleasant bedroom, and a general sitting-room which can boast of quite an extensive view from its windows; it has also a long book case which, like the one in the corresponding "nurse's sitting-room," is rapidly filling with a variety of volumes, gifts from different friends; the fine prints which adorn the walls of both rooms are also donations. The furniture and the colouring of these rooms are particularly pretty and tasteful, and the tables are covered with daily and weekly papers, magazines, &c., which ought to go a long way towards enabling nurses to escape that narrow-mindedness which is acknowledged to be a snare in their career. A valuable library of medical volumes has been collected, also by the liberality of outside friends. It is good to notice that the many comforts, nay! luxuries, have been amassed in this pleasant way without any taxing of hospital funds. The Sisters' rooms are situated away from the wards, and consists, in each case, of a good sized and prettily furnished apartment, with one end curtained off to serve the purpose of a bedroom. We feel sorely tempted to touch on other characteristics of this hospital, where so many praiseworthy innovations have found place within the time-honoured walls, but we remind ourselves that we are dealing now only with "the housing" of nurses, and other subjects are without our present province.

### Zenana Bible and Medical Mission.

### 40th ANNUAL MEETING.

The annual meeting of this society was held at Prince's Hall, Piccadilly, on April 27th, under the presidency of Lord Reay. Lord Kinnaird, whose sisters have visited the different stations of the society during the past year, was present, Sir W. Muir, General Hutchinson, General Mead, and many others. The progress which we must especially note during the past year is that of the increase of the work as regards the care of the sick. A new hospital has been opened at Lucknow, in memory of the late Lady Kinnaird, and already the number of patients has been so great that it has been found necessary to sanction the building of a new wing. Miss Mead is in charge at Lucknow. The patients treated at Benares, Lucknow, and Patna, counting in and out and home patients, number 8,904. There are now 47 lady missionaries, 26 assistants, and 203 native teachers and Bible-women. A new medical mission has been opened in Patna; early in 1891 Dr. Grace Mackinnon and Miss Gregory started work there, and in seven and a-half months they have treated 1,800 patients. The staff is quite inadequate for the enormous demand owing to the large tract of land the district covers, and at present the bungalow secured for the workers is very small, and certainly the illustration in the report does not lead one to suppose it is either comfortable or convenient. The Benares Hospital had to be closed for a short while owing to the death of Miss Wright and the absence of another lady on furlough; one special arrangement at the Benares Hospital is interesting to English readers, namely, the Purdah ladies' private wards; they are quite shut off from the rest of the hospital, and we must congratulate the Mission on the successful manner in which they seem to get hold of the high-caste women, for caste is the great barrier in India to progress of any sort.

At Ajoudhya, a Hindu city formerly of some importance, but destroyed by the Mussulman invaders, a dispensary has been opened, but at first the number of the patients was a little disheartening owing to the prevailing impression that medicine made up by a Christian has the effect of turning the patient Christian too!

Early in the year at Lucknow Miss Mead, with the assistance of Miss Haskew, performed Porro's operation on a Hindu woman quite successfully, and the child is living and the recovery is successful. This success made quite a stir among the friends and others who knew of it, and Miss Mead must feel very thankful, for a favourable result like this does more to admit the missionaries' ladies to the native women than it is possible over in England to credit.

We should like to hear of some more money finding its to the Secretary, Miss Hamilton, 2, Adelphi Terrace, Strand, for it must be remembered that the medical mission is only a part of this energetic society's work. They are doing a great deal towards education, and not only do the mission ladies teach the children who come to their schools, but, the force of example being as strong in India as elsewhere, the high-class Hindus are establishing schools where the future wives of their sons will receive a good education. Lord Reay gave personal testimony to the genuine enthusiasm of the workers he had met in India, and his remarks as to the result of the work must have been very gratifying to the enormous meeting of the friends of the mission whom we saw at Prince's Hall. A subscription at the meeting amounted to £240. The receipts for last year were £16,687, and we may mention that the Committee is anxious to raise the income to £20,000.

### Facts and Foundlings.

WITH the report of Dr. Sykes fresh in our minds, it is something of a surprise to find the Sunday morning service at the Foundling Chapel was well-attended by visitors, in some cases accompanied by several children, and also that two or three nurses in uniform were amongst the congregation. After the customary stroll through the museum and adjacent rooms, they found themselves confronted with notices to the effect that other portions of the building were closed to the public "until further notice," though why there should be more danger in keeping company with the children in their dining-hall, than in previously sharing with them for an hour and a-half the air of an ill-ventilated chapel, may well puzzle an unofficial mind. Perhaps it was as well that no explanation of certain doors being closed was volunteered, or some of the parents who were overheard consoling their young people for their disappointment at not being able to "see the dear little boys and girls" eating their Sunday dinner, might have returned home themselves sadder and wiser. Now that modern schools issue certificates that have to be returned signed before the readmission of the pupils, and which must solemnly assert that the boy or girl therein referred to has not been near any case of infectious disease, however light the nature thereof, we fail to find justification for visits paid by children to an institution where ten or eleven cases of scarlet fever are being nursed at present, even although the newer cases are now immediately removed to a fever hospital. We do not imagine that even the *Lancet* is so universally read that everyone can be already aware of the illness at the Foundling, although some reference to the fact has found its way into other papers; but we strongly maintain that in common justice to public safety, a notice should have been placed outside the building stating the presence of fever therein, and so giving fair warning to strangers of a possible and an avoidable danger.

### Registration of Midwives Bill.

"A COUNTRY Lady" sent a sensible letter to the *Morning Post* last week, with which, to a large extent, we agree. She writes: "No half or three-quarter educated midwife could take the place of a surgeon. The education received should be in this department the same as the surgeon's. In this special branch the same course should be pursued, the same examinations passed. If an Act is passed sanctioning the registration of midwives who are less educated and instructed than surgeons, the women will be the sufferers, though the surgeons would be gainers, as they would be relieved from ill-paid and ardous duties. These midwives should occupy the position of dentists. No one is compelled to employ a dentist, but dentists are generally employed. It would be unjust, however, to punish the neighbourly blacksmith who, in an emergency, operates on his friend's mouth with his pincers. There are clusters of cottages, two or three together, where no aid can be got but that of a neighbour. Punish the neighbour for officiating, and no aid will be had at all in future. If midwives as skilful as surgeons are established in market towns their aid will be sought for. If they are less skilful than surgeons their aid will not be sought." The Bill as it stands may not be all that could be desired, but its opponents seem to ignore the fact that its passing would bring us a great step on the road, whereas at present we have no enactment on the subject. There are no doubt many difficulties to be contended with, and many suggestions which could be made to further increase the standard of Midwifery, such as the question of increased length of training, but let us be thankful that the word "midwife" bids fair soon to become a title denoting a definite qualification, instead of being a mere cover to ignorant and greedy women, and a snare and delusion to those who call for assistance. We are glad to hear that already a good deal of evidence has been tendered relative to the Bill.

### Among the Institutions.

**Bristol Nurses' Training Institution.**—The annual report shows this institution to be in a flourishing condition. In 940 weeks' employment the earnings amounted to £2,290. Let us hope the Committee subscribe to the Pension Fund. We are glad to hear that fifteen of the nurses have joined on their own account.

**Burton-on-Trent.**—Here is a good example; will others please copy? The Friendly Societies' Association have handed over the sum of £33 18s. 9d. to Mrs. Bridgeman, the Hon. Secretary of the District Nursing Institution for the Poor. This sum is the result of a concert, and goes a long way towards the provision of a third night nurse, who has already begun work.

**Nurses' Home, Victoria Infirmary, Glasgow.**—The 19th of April saw the opening of two new nurses' homes in Glasgow. The Victoria Infirmary nurses have now an ideal dwelling place, with hot water, heating apparatus, and electric lighting. The furniture, which is the gift of Mrs. Renny Watson, is in every particular as comfortable and suitable as can be imagined, and the nurses have reason to be very proud of possessing so many good friends. The corridor which leads from the infirmary to the nurses' quarters has glass roof and sides, and is filled with plants. A silver key was presented to Mrs. Renny Watson, and in the recreation room she declared the home to be open.

**The Mary Orrell Higginbotham Memorial Home for Nurses** in Bath Street, Glasgow, was formally opened on Tuesday by Sir James King. It occupies the sight of the dwellings, which have been re-modelled at a cost of £2,600, exclusive of £3,250 paid for the premises. It will accommodate 82 nurses. The staff consists of two Superintendents (one for each department), 43 private nurses, 15 district nurses, 9 assistant nurses, 9 probationers, and 4 domestic servants, and as every nurse is now out, excepting three who are recruiting, the need for this large staff is fully proved. Mrs. Higginbotham, in whose memory the new home is, for many years devoted herself entirely to the Glasgow Sick Poor and Private Nursing Association.

**Belfast Society for Providing Nurses to the Sick Poor.**—During the past year the nurses paid 29,387 visits. We are glad to hear that the income met the expenditure; but this favourable state of things is due to the donations which were sent in by different friends as they became aware of financial straits. Mrs. Lindsay and Mrs. Charters, two ladies who did untold work towards starting and supporting the society, have died during the year; their loss is greatly felt. Mrs. Lindsay left £1,000 in her will to the funds. Two valued nurses (one of eighteen years' service, and the other of twelve) have had to resign owing to ill-health. A District Matron has this year been added to the staff. The younger nurses have joined or are joining the National Pension Fund, and the Committee is increasing the Superannuation Fund for the older nurses. The Corporation of Belfast is making great strides in sweeping away the unhealthy courts and alleys which were so numerous in the city. This will be of immense assistance to the nurses' work, who were terribly handicapped by the insanitary surroundings of their patients. We should like to hear of more Belfast firms becoming regular subscribers; the heads can scarcely realise, we should say, the amount of suffering among their workmen which is alleviated by this society.

### Everybody's Opinion.

*[Correspondence on all subjects is invited, but we cannot in any way be responsible for the opinions expressed by our correspondents. No communications can be entertained if the name and address of the correspondent is not given, or unless one side of the paper only be written on.]*

### SOMETHING MORE ABOUT HOLIDAYS FROM A NURSE.

"Nurse Blue" writes: I am a nurse, and I read The Hospital; and, having noticed what has been said about nurses and their holidays, I would like to tell fellow-nurses how I and two friends (both nurses) spent a holiday last September. I was a private nurse at the time, and No. 2 was a Queen's Jubilee Nurse from a busy town, who said she felt she must get away to see some hills and some sheep. Then No. 3 had done nothing since leaving hospital work. At first it was proposed that we should go to some pretty place, and just wander about, or be lazy, as we felt inclined, but after much discussion it was arranged that we should go a walking tour, and this we carried out. I am not to tell you where we went, but we saw the Queen and one of her castles, so perhaps you can guess. In one week we walked about seventy miles (does it not frighten you?), and when it was over we were wishing for next year, and all declaring we would do it again. It would take up too much time and space to tell of all the fun and enjoyment we had. Strange to say a wet day caused endless fun. It was too absurd to have to go to bed at two p.m. that your clothes might be dried. (I should tell you they were clothes we could not spoil.) Now one word about all the kind people we saw on our tramp, landladies of hotels, &c. They were all extremely kind, and seemed to take great interest in us, and were very moderate in their charges. We remarked when one old lady presented her account that she had charged less than she said she would, and the answer we got was something like this: "Yes, you's ladies, and gives no trouble"! I can answer for myself that the road we tramped is so firmly printed on my mind's eye that I can recall almost any bit of road at will, and a lovely road it was. I will close my little note by wishing some other nurses may have as happy a time as we did.

### NURSES' HOLIDAYS.

"A Lover of Nurses" writes: In answer to your correspondent's very sensible letter on "Holiday Experiences," in your issue of April 9th, I beg to suggest a capital fortnight's trip, by no means difficult to carry out by those who are fairly good sailors and have a little go and enterprise left in them wherewith to enjoy "variety from the usual routine." This is, as she observes, the one thing to be aimed at in a nurse's holiday. Let me advise a sea-trip down the channel to Cork and back. The first-class return ticket costs 30s. only; you start at eight from Miller's Wharf, Lower East Smithfield, on a Thursday morning, and arrive on Sunday morning at Cork. This is not an attractive place, and I should advise holiday makers to go on third-class by rail 26 miles to Youghal Bay, a primitive sunny little fishing village, where everything is wonderfully cheap, and the people themselves simple and hospitable. Salmon-fishing is the staple trade there, and sea bathing is possible; a bed-room can be had from 7s. a week. A friend of mine, a tired out London mission worker, following my example, went in this way and had nearly a month's complete holiday for less than £4; two friends going together need not spend quite so much as one going alone. Some little time back the doctors were all recommending a cruise down the channel in preference to a trip abroad as the quickest way of taking in a stock of health wherewith to meet the severe strain of modern life, and so I found it. It would be possible to go and return within a fortnight if need be. Starting on Thursday morning from London, and arriving on Sunday morning at Cork, you would have a week at Youghal, and then return by the Saturday-afternoon boat from Cork, reaching London again on Tuesday morning. A little portmanteau for luggage, some nice books and needlework, a fold-up chair, and plenty of wraps, are all that are necessary. Messrs. J. Hartley, 19, Leadenhall Street, are the Cork Steam Packet agents (see page 541 of April Bradshaw). Apartments might probably be secured beforehand at Youghal by negotiations with the post-office, but I should myself think it quite eligible to take my chance on arrival. Remembering the adage "Who helps a nurse helps many more," I hope this letter may be of use to some pair of poor tired-out, spiritless nurses with hardly energy left wherewith to utilise a holiday.

### A DIFFICULT CASE.

Messrs. E. Hopgood, 23, Windsor Road, Ealing; J. Morgan, 16, The Mall, Ealing; and F. E. Tuckey, Ely House, Oxford Road, Ealing, Past Presidents of the Ealing Philanthropic Institution, earnestly desire to commend a most deserving case. A young man, full of promise and intelligence, had the misfortune, at the commencement of his career, arising from overwork, to suffer a mental affliction, necessitating his removal to the county asylum. We have the pleasure to say that he has been returned perfectly cured, and it is now proposed to start him in a suitable business here in Ealing, where he has spent his young life and has earned the esteem of all who know him; for this purpose we venture to ask for some monetary help. We shall be glad to answer any enquiry, and gratefully acknowledge all subscriptions.

### Where to Go.

The New Gallery.—It is always a relief to the Londoner who cares something for current art to feel that the picture exhibitions are ready to open their doors once more, and a pleasant solution of where to spend a spare afternoon is offered to him without further effort. We will just point out one or two of the pictures at the New Gallery, whither we advise nurses to go before the crush of people begins. Among the portraits here, that of Paderewski by Mr. Alma Tadema, is one of the finest and most interesting. Princess Louise also sends a study of the famous Polish pianist, which is, however, hung in another room. The portrait of Mrs. Marsden Smedley, by Mr. W. B. Richmond, is a picture as well as a portrait; it is a clever full length figure, with groups of rhododendrons and ferns, a beautiful piece of colouring; while the only portrait which interests among those sent by Mr. Shannon is his one of Miss Wordsworth. Mr. Watts has the place of honour in the Central Gallery with his portrait of Walter Crane. Mr. Alfred Parson's daffodils and river bank are charming, as is also Mr. David Murray's "Hampshire Haying." Space forbids our mentioning the many other good things of this exhibition, excepting Mr. Tadema's "Dreaming," which no visitor to the New Gallery can afford to overlook.

### Notes and Queries.

### Queries.

*Microscope.*—Will some reader give me the composition of the black or white enamel used for ringing microscopical slides?—*A. Wightwick.*

*Middle-Class Motherless Children's Home.*—Can any reader tell me if there is a home for motherless children of the middle-class where they are brought up and educated free of expense. The father of the children I am interested in is in very reduced circumstances.—*Sister H.*

### Answers.

*Change of Work.*—(*F. R.*) Get the *Hospital Annual*, or look every week in our advertisement columns.

*Mineral Baths.*—(*N. C. B., Bath.*) Royal Mineral-water Hospital would do for you most likely. Write to the Secretary and state your case, and that you are willing to pay. If you cannot get on, write again.

*A Midwive's Library.*—(*Mrs. Power.*) Barnes' "Manual of Midwifery," Playfair's "Midwifery," Cullingworth's "Manual for Monthly Nurses," Lewis's "Theory and Practice of Nursing." You can procure this paper at "Smith's" Station Book-stalls. No, no agency.

*Convalescent Case.*—(*Contributor, April 9th.*) Your case might be taken by the Merchant Taylors' Company's Convalescent Home at Bognor. Apply to the Secretary, The Hall, Threadneedle Street, E.C. We will find out about the home you enquire for. We are sorry your query has received no reply from correspondents:

*Further Training.*—(*A Nurse, No. 3.*) Will you another time choose another *pseudonym*, as there are so many correspondents who sign themselves "Nurse"? We suppose you really wish to complete your training, is not that what you mean by polish? Why not stay where you are? Write fully to "Nursing" at this office, and we will tell you what will be your best plan. What training have you had?

*Wanted, a Certificate.*—(*Enquirer, No. 2.*) You must have active training in a hospital to gain a certificate. There is luckily no such system as you describe, or the public would need a great deal of pity. Do not try to be a nurse as you say you are not strong. You could gain knowledge by going through the St. John Ambulance course, which would be very useful to you in private life, but a *trained nurse* is a very different matter. This sounds hard advice, but do not listen to any other.

*Sister Hilda.*—Incurable children are received, and are well nursed and cared for, at St. Lucy's Home, Hare Lane, Gloucester. There is a large garden where, in the summer, the patients are carried out and placed upon couches:

### Four Months in a Hospital Ward.

### A PERSONAL EXPERIENCE IN A PROVINCIAL HOSPITAL.—(*Continued.*)

### Forgotten Requirements.

It has been said that constant attendance upon the sick, especially in hospital, hardens the nurse and destroys the natural feeling of sympathy with suffering we all experience more or less, and that the duties come to be performed in a purely perfunctory manner; it is not so, I saw numberless instances during my sojourn in that ward of keen sympathy on the part of the nurses, only with an entire absence of hysteria, and therein lies the great superiority of a trained over an amateur nurse, as any patient who has had an experience of each will tell you. To me was brought "light meat diet" with bread and half a pint of milk, but where was the knife and fork? then I learnt that I ought to have brought these with me, and also a spoon, some butter and sugar and tea, and a towel and soap. What was I to do? "Oh!" said the cheery nurse, "we'll soon get over that difficulty," and she quickly brought me a knife and fork which she had borrowed somewhere, and suggested that doubtless one of the patients would lend me the other requirements until I could communicate with my friends. The meal over —a very few minutes sufficed to accomplish it—the nurses came round for the plates and cups, and took them away to the ward scullery at the end of the corridor. Every ward has its own scullery; and then they brought brooms and swept up any crumbs, passed a quick, neat hand over our coverlets, and once again we were in "apple pie" order. Then comes the medicine: this is always standing upon the patient's locker, and the nurse comes round with a measuring glass and a small basin of warm water and a towel. She makes her journey round the beds, and carefully washes and dries the glass after each patient. At this entertainment no one is allowed to "shirk the bottle," no dallying, nice or nasty down it must go; here and there you see a wry face, but rarely a murmur, and then only when a patient is more or less delirious, so perfect is the discipline of the ward. The main business of the day is now over.

### We are Spared an Infliction.

The house doctor comes round at nine o'clock in the morning, and the honorary physician for the day at about eleven o'clock. There is no medical school, so we are spared the infliction of being made the centre of a class of students. It must be rather depressing to be the subject of a lecture, and hear an unfavourable prognosis of one's own case, but perhaps its technicalities, like the notes on the "bed boards," would shroud its meaning from any lay perception; and after all, it is the only return a hospital patient can make for the gratuitous and invaluable benefits of which he is here the recipient. The bandaging, poulticing, painting, blistering, and small surgical operations, are all performed in the first half of the day, and keep the patients more or less on the *qui vive*, so that after dinner there comes a refreshing quiet that invites repose, and those whose pains will let them, fall gently into blessed oblivion of their troubles.

### Our Amusements.

This, too, was the time when nurses and probationers from other wards would sometimes look in upon us, all of them kindly and genial, treating us as so many brothers, and never losing an opportunity of interesting or amusing us. One nurse would bring a cage of tame canaries, over which she had perfect control: the birds would perch on her shoulder, eat crumbs out of her hand, and hop in and out of the cage at her will. Another would introduce to our notice a splendid old tabby cat, that could do anything short of talking, and fiercely resented the slightest approach of a strange hand towards its mistress; it could jump like a greyhound, and would give us a steeplechase in miniature to perfection. A milky white Pomeranian dog was the companion of another of the nurses, and well up in all the usual "doggy" accomplishments. This gentleman was evidently no probationer, from the cool manner in which he would trot up and down the ward with his weather-eye open for patients' sugar. Sometimes a nurse would bring in a large musical-box, and we could then take our siesta to the lullaby of "Home, Sweet Home" or "Auld Lang Syne"; and last, but I cannot say "not least," for she was veritably the smallest nurse in the hospital, would come a tiny probationer, whom we called the "baby nurse," and play game after game of draughts with such of the patients as were able and inclined to this form of recreation.

### "Johnnie."

One little fellow, Johnnie, who died in our midst, was very much attached to this nurse, and when he was gone her face was a shade sadder, I thought. She used to say that her favourite ward was the children's, and I hope, ere this, she has found her proper sphere on the establishment of some Children's Hospital, for which she was pre-eminently fitted, for she loved children and they loved her. Strong, healthy men, with sound bodies and minds, may smile at the simplicity of the amusements and devices to beguile the weary hours of suffering which I have described, but it is just such light fare as this which is best calculated to catch the attention of minds weary and weakened by prolonged illness, and so divert the thoughts, if only for a few moments, from the morbid channel of self —an effort which, if successful, is quickly followed by a manifest improvement in tone. I will take this opportunity of saying a few words about the nurse who, to my eyes, was the central figure in this family of Samaritans, I mean our charge nurse, who returned from a holiday soon after my advent. She was the oldest member of the nursing staff, and well worthy to be called the "central figure," for although of severe mien, a strict disciplinarian, and almost a martinet where the probationers were concerned, yet deep down beneath this stern exterior was a warm spring of human kindness and sympathy.

### "A Past Mistress."

I must frankly confess that on her first coming into the ward I was not favourably impressed with this "past mistress" of the nursing art. How quickly was this adverse impression dispersed! Before the first week of her return was over I saw unmistakably that her whole soul was centred in the interests of the patients. With her, economy in management might be an excellent thing, the comfort of the probationers a praiseworthy object, rules and regulations necessary evils, but the patient stood before all and everything. She was jealous of the slightest interference with her department, and resented in unmistakeable language any carelessness or neglect on the part of her subordinates towards those sick men under her care. If a patient fancied soda-water or any other special item, it was always this "austere" nurse who would get the doctor to put it on the "bed board," and when she saw a patient shirking his food, she never rested until she had found something that he could eat. I have known her take to a patient who from sheer weariness was refusing his meals, a cup of tea and a slice of dry toast, which she had prepared herself in her own quarters, all daintily set out in pretty china service, and gently coax him to try and take it. It must have been irresistible, for the man did accomplish the fragile meal, to his evident benefit. Even after a patient had left the hospital her interest in him did not cease, for to my knowledge she has visited and taken comforts of her own providing to the squalid home of some poor fellow, who though cured, has yet months of strength-building to accomplish before he will be capable of a day's work.

*(To be continued.)*

### A True Story of Flowers.

*(Concluded from page xxxvi.)*

Then came the time of roses. Oh, the glory of these roses! "Summer is nearly here," they told her. "Can you hear her coming, sweet?"

The Princess listened; she could not hear as well as the roses, yet she trusted them, and the news gladdened her heart. Surely summer would make her well.

"When I get out again," she said; but no one answered her, and she thought that strange.

"Summer is bidding us show our love for the King," the roses went on. "We are bursting our buds with happiness, we scatter our petals in the wind, our buds unfold to tell his story."

"Yet though these fade
From thy dead leaves, let fragrance rise
And teach the maid,
That goodness time's rude hand defies,
That virtue lives when beauty dies."

"I know it, I know it, I know it," answered the throstle from the window. He had been eating a strawberry on the ledge. He did not sing so much now, but he loved the Princess all the same. He was always interrupting the flowers, and they did not like it, they liked to be the only flowers to tell the beautiful story in that sick room. They were never jealous of each other, only of the birds; though once the Princess caught the beautiful Malmaison carnations looking a little haughtily at a rude bunch of red dahlias and marigolds; but when they heard the story of that bunch, they never showed a sign of pride again. The dahlias and marigolds brought the Princess as much love as the carnations did; no message was so humble but that she valued it the more.

A glorious morning broke when all gathered round that bed, and the angels folded their wings and hung their heads, for the King was there in all His glory. White flowers were by the little white cross on the fair cloth, and the roses blushed as the Body and Blood of the Lord touched the lips of each. Only three white arum lilies dared to look up, pointing a golden finger heavenward. . . "O, Lamb of God, that taketh away the sins of the world" . . . then only a sound of weeping.

The throstle was afraid to sing, and the flowers hardly whispered for a long time after.

So time passed, and the flowers marked the way. Sweet peas and peonies, mignonette and cherry-pie, treasures of burrow roses, each came in their turn to brighten and breathe hope. But the song would be endless if even the angels sang of every blossom which bloomed in that room.

Oh, the flowers! The flowers! Could anyone have lived through the eight long weeks in the great city without them, when the fight was hardest? When even the Princess's courage almost gave way, when the Angel of Death stood waiting. Roses, sweet roses! But for thee the struggle might have been given up; but for your beautiful whisper of "patience" the pain may never have been borne.

A bowl of heather brought wafts of fresh air from the moors, and visions of blue skies and mountains were woven in the dreams of the Princess that night. Blue harebells from the Lakes rang a peal of gentle gladness, and loose yellow roses nodded their heads and kept time in a fairy bower of tender, soft green maidenhair.

There was a flower of the rare buck-bean . . . Orange alstrœmeria, white clove scented pinks . . . an amethyst bowl of peonies with purple hearts flushing among brown leaves and waxen heath . . . . . an endless wealth of all that was sweet and beautiful, and then a little bunch of blue forget-me-nots from a river side.

"That blue and bright-eyed floweret of the brook,
Hope's gentle gem, the sweet forget-me-not."

Yes, hope came, and . . . . . the Princess opened her eyes to find herself in the country. She thought it must be a dream at first, but the flowers told her she was really there!

A glass full of La France and one of blood-red roses, a blue bowl of white sweet peas like pearls with their gentle sea-green tendrils, and soft-coloured carnations were awaiting her coming. "Welcome," they sang, for the King had whispered the secret, and they listened, and when she looked there was no need for others to tell her she had left the Great City behind: there was no need to tell her "God made the country." The birds sang the song she was trying to sing, and the angels understood and carried the song heavenward, even though she was too tired to put it into words. She knew well that the King hears always before we speak, words are never needed to make Him understand. Love interprets the heart's least thought. "Before ye speak I will hear, saith the Lord," and this satisfied the Princess. A sweet content overshadowed that little world; she was coming slowly back to life. All the love and care which had been unceasingly and untiringly lavished upon her would have its reward. The mother's patient love had won the victory of prayer, and the flowers had not bloomed in vain.

Surely she had found in her pain—God. Surely the long suffering had verily and indeed been a pathway of flowers.

"Oh, what a beautiful world!" the Princess exclaimed when she saw the trees, and the sky, and the green grass. "Oh, what a beautiful world!"

Does anyone know that feeling? After being in a prison-house of four dull walls—where it is hard to believe the world is fair—to come out into the sunshine which blinds and dazzles one, for the soft green shines like a sea of emeralds and the sun as burnished gold. Those who have never experienced this cannot understand. . . . there are some who can!

Looking back now, it is only the flowers and the love which the Princess remembers. The King told her she had her task to finish, that there was one who had need of her, so she obeyed Him. Only the world will never be the same world again; she can never doubt, or trouble, or wonder. Oftentimes when the stars peep out to see if the world sleeps she prays to the King that her story of flowers may find an echo in many hearts.

"Wondrous truths, and manifold as wondrous,
God hath written in the stars above;
But not less in the bright flowerets under us
Stands the revelation of His Love."

This is the song the Princess oftentimes sings in the sunlight—

"You who have flowers, oh! share them with the sick and suffering; you who have flowers send them to the sad and sorrowing. Do not tarry on the way but send them, and reap blessings for the kindly deed. They are the King's jewels; each blossom blooms in answer to His will, and surely for some good purpose. They are entrusted to your care, a precious trust for the sake of others. You who have health may not understand so well, but I have suffered . . . perhaps that I might tell you what a blessing flowers are. Send them, for I ask you. One flower may breathe Hope, another Courage, another Patience—you can never know. . . . Only send them."

The Princess could not finish her song, for someone was calling her. . . .

. . . But the angels took it up, and it will be sung through all eternity.

## Figures and Tables

**Figure f1:**